# Intraorbital Steroid Injection for Active Thyroid Ophthalmopathy

**DOI:** 10.18502/jovr.v15i1.5948

**Published:** 2020-02-02

**Authors:** Abbas Bagheri, Mohammad Abbaszadeh, Shahin Yazdani

**Affiliations:** ^1^Ocular Tissue Engineering Research Center, Shahid Beheshti University of Medical Sciences, Tehran, Iran; ^2^Ophthalmic Research Center, Shahid Beheshti University of Medical Sciences, Tehran, Iran

**Keywords:** Graves, Lid Retraction, Orbital Inflammation, Proptosis, Steroid Injection

## Abstract

**Purpose:**

To evaluate the effect of orbital steroid injections in patients with active thyroid ophthalmopathy resistant to or dependent on systemic steroids, or with complications related to systemic steroid use.

**Methods:**

This prospective non-comparative case series includes 31 eyes of 17 patients with active thyroid ophthalmopathy and clinical activity score (CAS) of 3 or more, without compressive optic neuropathy or overt exposure keratopathy. All subjects had a history of previous systemic steroid use (with steroid resistance or dependence) or had developed complications related to steroids. A combination of steroids including triamcinolone acetonide 20 mg and dexamethasone 4 mg was injected in the upper and lower retroseptal orbital spaces three or four times at one-month intervals. The patients were examined periodically after each injection and at least three months after the last injection.

**Results:**

Mean pre-injection CAS was 5.2 ± 1.3 which was improved to 1.6 ± 1 after the fourth injection (*P* < 0.001). Upper and lower lid retraction improved in 100% and 68.2% of the affected eyes, respectively. Strabismus completely resolved in one of five affected patients and the most significant improvement was observed in supraduction. Mean improvement in exophthalmos was 1.2 ± 1.1 mm. Visual acuity did not significantly change after the injections. Eyelid ecchymosis and/or subconjunctival hemorrhage was observed in 7.1% and intraocular pressure rise occurred in 8.8% of eyes.

**Conclusion:**

Orbital steroid injections can be used for the treatment of active thyroid ophthalmopathy when the patient is resistant to or dependent on systemic steroids or has developed complications of systemic steroids.

##  INTRODUCTION

Graves' disease is a common autoimmune disorder associated with thyroid dysfunction which presents with various metabolic and immunologic disturbances.^[1,2,3,4]^

Orbital tissues become involved in up to 50% of patients with Graves' disease. Because of the compact arrangement of tissues in the confined space of the orbit, variable degrees of inflammation can lead to serious complications such as proptosis, strabismus, corneal exposure, and compressive optic neuropathy. Drastic signs are fortunately uncommon and are observed in less than 5% of Graves' patients.^[1,2,3,4]^ Ophthalmopathy in Graves' disease often lasts for one–two years and the peak of disease activity precedes the peak of disease severity by six months.^[4]^

In mild forms of Graves' ophthalmopathy, conservative management such as the use of lubricants, administration of oral selenium, control of thyroid dysfunction, and cessation of smoking may suffice;^[3,5]^ however, moderate to severe cases may require systemic steroids via oral or intravenous routes.^[6,7]^

Due to resistance to and dependence on steroids, or complications related to systemic use of steroids including gastric ulcer, osteoporosis, activation of tuberculosis, weight gain, increased glucose levels, and systemic hypertension,^[8]^ some authors have suggested local injection of steroids.^[9,10,11,12,13,14,15,16,17,18,19,20]^ Steroids may be injected locally in the subconjunctival space^[9,10,11,12]^ or retroseptally within the orbital space,^[13,14,15,16,17,18,19]^ and have been shown to entail lower complications than systemic steroids.^[20]^

Herein, the authors present the results of intraorbital injection of steroids in patients with active Graves' ophthalmopathy resistant to or dependent on steroids or subjects who had developed serious complications due to systemic steroids.

##  METHODS

This prospective interventional case series was approved by the Scientific and Ethics Committee of the Ophthalmic Research Center at the Shahid Beheshti University of Medical Sciences and adhered to the principles outlined in The Declaration of Helsinki and written informed consent was taken from all the patients.

In the current series, we included adult subjects from January 2017 to March 2018, affected by thyroid ophthalmopathy with disease activity and severity score of 3 or more who had received oral or intravenous steroids. All patients were resistant to, or dependent on, steroids or had developed systemic complications such as hyperglycemia, hypertension, gastric ulcer, major depression, unacceptable weight gain or moon face, and the patient was unwilling to continue systemic steroids.

The exclusion criteria consisted of compressive optic neuropathy, glaucoma, active periocular infections, history of orbital radiation and anticoagulation therapy. All patients were instructed to discontinue systemic steroids at least four weeks before consideration for the new treatment modality. Any patient who failed to adhere to the treatment schedule up to the end of the study period was also excluded.

After obtaining a complete history related to the disease, a comprehensive eye examination was performed including slit lamp examination, funduscopy, and tonometry. External examination included evaluation of eyelid condition and severity of lid retraction. Ocular deviation was assessed using prisms to compensate for any diplopia. Limitations in eye movements were documented as duction limitations. Exophthalmometry was performed employing the Naugle exophthalmometer. To evaluate the activity and severity of Graves' disease, we used the “Clinical Activity Score" (CAS) that includes 10 grades and the “NOSPECS" method that consists of 6 grades, respectively.^[21,22,23,24]^

All intraorbital injections were performed by the first author (AB) in the operation room under stand-by anesthesia with systemic monitoring by an anesthesiologist. After sterilization of both lids with ethyl alcohol 90%, triamcinolone acetonide 20 mg (Exir, Iran) plus dexamethasone 4 mg (Darou Pakhsh, Iran) were prepared in a 2 ml syringe for each injection site and were injected into both superior and inferior retroseptal orbital space from the middle of the orbital rim using a #26 gauge needle 2.5 inches in length while the globe was protected by the free hand of the surgeon. The volume of injection for each site was 1 ml and a total of 2 ml mixed steroids were injected inside the orbit. After the injections, ocular massage was performed and intraocular pressure and vision were checked five minutes later; the patients were discharged if the injection was found to be uneventful. In all patients, the injections were repeated at least thrice at one month intervals. If residual inflammatory signs were present, a fourth injection was also added at the same interval. All examinations were repeated the day after the injection, and one and three months after the last injection; any complications were documented.

Changes in the disease severity and activity were evaluated separately. For disease severity, any improvement from higher to lower categories of NOSPECS was defined as success. For disease activity, a decrease in CAS to 3 or less was defined as success. For proptosis, a decrease in exophthalmos of at least 2 mm; for lid retraction, a decrease of at least 1 mm; and in case of ocular motility, any improvement in duction limitations was defined as success. If the disease became quiet and stable at least three months after the last injection, reconstructive or cosmetic procedures including orbital decompression, strabismus surgery or lid surgery were performed as required.

Data analysis was performed using SPSS software version 25. For the description of findings, we used percentage, mean, standard deviation, mode, and range. For the comparison of study parameters, we employed the paired *T*-test for intragroup analysis; to compensate for the use of fellow eyes from the same subject, we used Generalized Estimating Equation (GEE) analysis. Spearman correlation was used to evaluate the relation between parameters.

P-values < 0.05 and confidence intervals of 95% were used to determine the statistical significance.

##  RESULTS 

Initially, 41 eyes of 22 patients were enrolled in the study; however, five patients were excluded from the final analysis due to non-adherence to the treatment protocol. The results reported herein are based on the data from 31 eyes of 17 patients. Patient characteristics are detailed in Table 1. Mean LogMAR visual acuity before the injections was 0.14 ± 0.1, which remained stable at 0.13 ± 0.09 LogMAR with no significant change (*P* = 0.3).

**Table 1:** Clinical and paraclinical characteristics of patients


**Variables**	
**Age**	Mean (range)	49.8 ± 12.2 (28–72 years)
**Duration of Graves' disease**	Mean (range)	7.3 ± 7.2 (9 months to 23 years)
	Numbers (%)
**Sex**	Male	11 (64.7%)
	Female	6 (35.3%)
**Smoking **	Smoker	9 (52.9%)
	Nonsmoker	8 (47.1%)
**Thyroid function when Graves' ophthalmopathy started **	Hyperthyroid	16 (94.1%)
	Hypothyroid	1 (5.9%)
**History of surgeries for Graves' ophthalmopathy **	Orbital decompression	4 (23.5%)
	Strabismus surgery	2 (11.8%)
	Eyelid surgery	2 (11.8%)
**Previous treatments for Graves' disease **	Antithyroid (Methymazole)	11
	Radiactive Iodine	3
	Thyroidectomy	2
	Plasmapheresis	1
**Present treatments for Graves' disease**	Antithyroid (Methymazole)	11
	Levothyroxine	3
Causes of using orbital steroid injections*	Resistance to systemic steroids	5 (%29.4)
	Dependence on systemic steroids	2 (%11.8)
	Complications of systemic steroids	10 (%58.8)
	Hypertension	7 (%41.2)
	Hyperglycemia	3 (%17.6)
	Moon face	2 (%11.8)
	Major depression	1 (%5.9)
	Flushing	1 (%5.9)
	Severe weight gain	1 (%5.9)
**Disease severity (NOSPECS)**	Score	
	< 3	0
	3	1 (%5.9)
	4	13 (%76.5)
	5	3 (%17.6)
	6	0
**Disease activity (CAS)**	Score	
	< 3	0
	3–6	28
	> 6	3
	
	
*Some cases had more than one cause		

**Figure 1:**
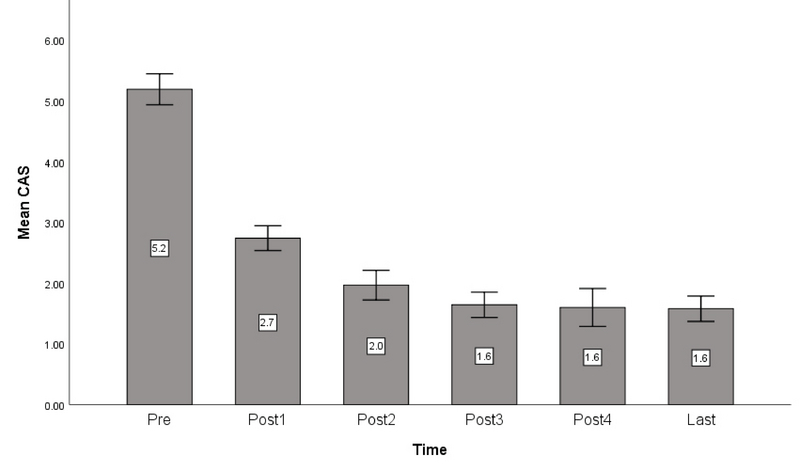
Bar diagram of changes in mean clinical activity score (CAS) before the injections up to the final examination.

**Figure 2:**
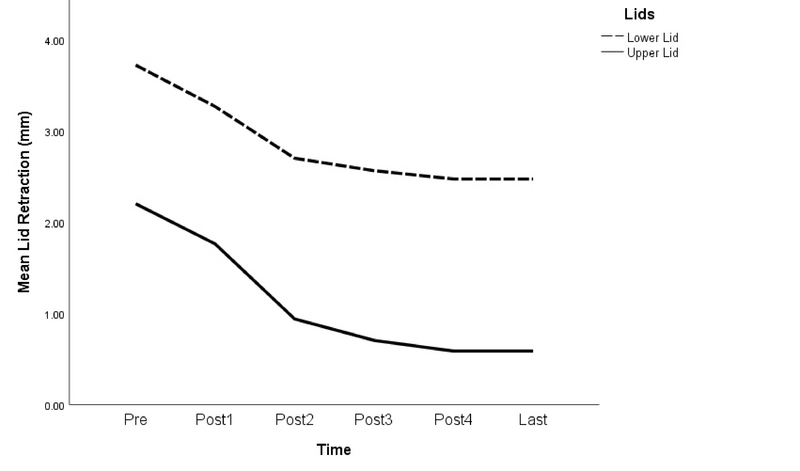
Line diagram of changes in mean lids retraction before the injections up to the last examination.

**Figure 3:**
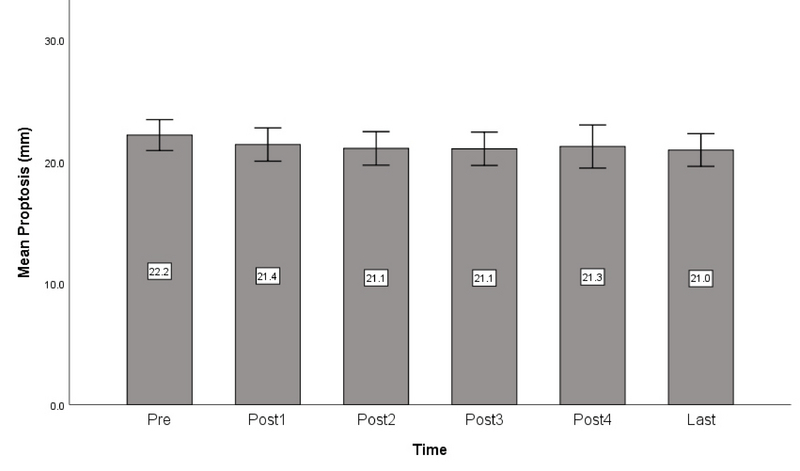
Bar diagram of changes in mean amount of proptosis before the injections up to the final examination.

**Figure 4:**
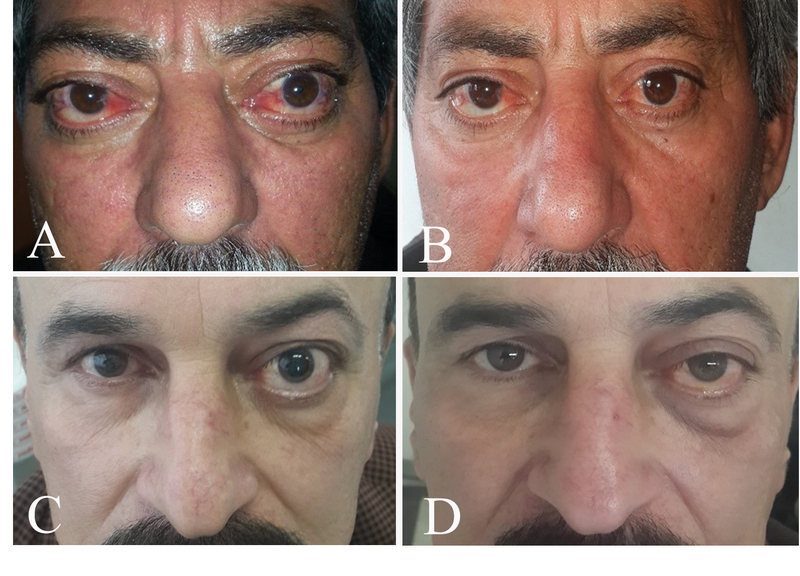
Two representative patients before injections (A,C) and at final examinations (B,D).

Before the injections, three eyes (9.7%) had stage 5 disease according to the NOSPECS severity classification that improved to stage 4; in the remaining cases, the disease severity remained unchanged (*P* = 0.9). The mean CAS before the intraorbital injections was 5.2 ± 1.3 that improved to 2.7 ± 1 after the first injection and to 1.6 ± 1 at the final examination. Changes in CAS scores were significant after every single injection (*P* < 0.001, *P* < 0.001, and *P* < 0.005 for the first to third injections, respectively), but not significant afterward. The most significant change in the CAS score was observed after the first injection [Figure 1].

Upper lid retraction was present in 17 eyes (54.8%), which was improved by at least 1 mm or more in all eyes (100%) at the final examination. Lower lid retraction was present in 22 eyes (71%), which was improved by 1 mm or more in 68.2% of affected eyes at the final examination. The mean amount of upper lid retraction before the injections was 2.2 ± 1.3 (range, 1–4) mm, which was reduced to 0.6 ± 1.1 (range, 0–3) (*P* < 0.001) at the final examination. The mean amount of lower lid retraction before the injections was 3.7 ± 1.9 (range, 1–9) mm, which was reduced to 2.5 ± 1.5 (range, 0–5) mm (*P* < 0.001) at the final follow-up. The amount of improvement was significant only after the first injection for upper lid retraction (*P* < 0.001) and after the first and second injections for the lower lid retraction (*P* < 0.001 in both cases) [Figure 2].

Five patents (29.4%) had strabismus prior to the injections that included two cases of esotropia, two cases of esotropia with hypotropia, and one case of consecutive exotropia in a subject with a history of twice weakening of both medial recti. The mean amount of deviation before the injections was 39.4 ± 29.8 Δ horizontal deviation (range 90 Δ ET to 15 Δ XT) with 54 ± 50.9 Δ (range, 18–90 Δ) hypotropia, which remained unchanged at 38.8 ± 30.4 Δ horizontal deviation (range, 90 Δ ET to 12 Δ XT) with 54 ± 50.9 Δ (range, 18–90 Δ) hypotropia (*P* = 0.4 and *P* = 1, respectively).

The mean amount of limitation in supraduction was –0.4 ± 1 (range, –4 to 0) before the injections, which was improved to –0.2 ± 0.9 (range, –4 to 0) at the last follow-up (*P* = 0.04). The limitation in abduction was –2.2 ± 0.8 (range, –4 to –1) before the injections, which remained unchanged at –2:00 ± 0.8 (range, –4 to –1) at the final follow-up (*P* = 0.2). Changes in adduction and infraduction were also not significant (*P *= 0.9 for both comparisons).

Exophthalmos was improved by at least 2 mm in 7 eyes (22.6%), and by 1 mm or more in 17 eyes (54.8%) at the final follow-up. The Mean exophthalmometry readings before the injections was 22.4 ± 3.5 (range, 16–28) mm, which was reduced to 21 ± 3.7 (range, 15–27) mm at the last follow-up. The mean decrease in exophthalmos was 1.2 ± 1.1 mm at the final follow-up as compared to baseline values. The decrease in exophthalmos was significant only after the first and second injections (*P* < 0.001 and *P* = 0.05, respectively) [Figure 3].

None of the changes observed in the study parameters including disease severity according to NOSPECS, disease activity according to CAS, lid retraction, strabismus, ocular movements, and exophthalmos had a significant correlation with age, sex, length of thyroid ophthalmopathy, and smoking [Figure 4].

After the injections, thyroid eye disease became inactive in all eyes; thereafter operations were performed in a number of cases including cosmetic orbital decompression in seven eyes (22.6%), strabismus surgery in four patients (23.5%), and eyelid retraction surgery in three eyes (9.7%).

Overall intraocular pressure elevation occurred after the injections in 10 out of 113 injections (8.8%), ranging from 21 to 32 mmHg and was controlled with topical medications after a few days. In a total of 8 out of 113 (7.1%) injections, subconjunctival hemorrhage or periorbital hematoma occurred, which resolved spontaneously after a few days. Other transient complications included ptosis in one case, hyperpigmentation over the injection sites in one case, and hypermenorrhea in another case, all of which improved spontaneously.

##  DISCUSSION 

The current study demonstrated that intraorbital steroid injections can be used to control inflammation and decrease disease activity in Graves' ophthalmopathy. These injections improve limitations in ocular motility, exophthalmos and lid retraction, and reduce the severity of the condition.

Oral or intravenous systemic steroids are the drugs of choices for active Graves' ophthalmopathy.^[6,7][25]^ When the condition does not respond to these routes of steroid administration, or if the patient becomes dependent on them or develops steroid-related complications such as systemic hypertension, hyperglycemia, gastric ulcer and obesity, alternative modalities of treatments may be employed. These include orbital radiotherapy,^[26,27,28]^ cytotoxic drugs^[29,30,31]^ or biologic agents,^[32]^ each of which entails shortcomings and potential complications.^[26,27,28][29,30,31][32]^

Local injection of steroids has been performed using a variety of methods for the treatment of Graves' ophthalmopathy. It has been used as subconjunctival injections to treat lid retractions,^[9,10,11,12]^ which not only led to improved vision but also decreased extraocular muscle thickness.^[9]^ Our treatment protocol that included retroseptal injections in both the superior and inferior orbital spaces significantly improved upper and lower lid retraction.

Authors using intraorbital steroid injections have reported different results. Khafagy^[16]^ reported reduced proptosis in 80% and improvement in ocular movements in 100% of their cases, while Kim and Jung^[15]^ concluded that orbital steroid injections had no effect on proptosis or ocular movements. In the current study, we observed a mean improvement of 1.2 mm in proptosis and also some improvement in ocular motility, especially in supraduction, although the angle of strabismus did not change significantly.

One of the most important parameters used for evaluating the efficacy of local steroid injections is the improvement in inflammatory signs. Bordaberry et al^[17]^ reported a reduction of CAS from 6.4 to 1.8 with this treatment modality and even improvement in compressive optic neuropathy in 66% of affected cases. Yakopson et al^[18]^ showed a decreased inflammation in 63% of their cases while Jung and Kim^[15]^ also reported a significant improvement in inflammation. In our study, CAS score decreased from 5.2 to 1.6, which indicates a significant reduction in inflammation.

Various injection intervals have been employed by different authors that include one week,^[13,14,16,20]^ two weeks,^[15,17]^ three weeks,^[12]^ four weeks^[9,11]^ and even longer intervals of about four months.^[10,18]^ We used four week intervals because the effect of triamcinolone lasts for four weeks,^[19]^ so this interval seems the most logical choice. Previous studies have reported a total number of injections ranging from one to twelve;^[9,10,11,12,13,14,15,16,17,18,19,20]^ we observed that the earliest injections are the most effective and the therapeutic effect decreases with repeating injections. In our patients, the decrease in CAS score was significant up to three injections, but not significant afterward. The effect on proptosis, lid retraction, and extraocular muscles were also significant up to two injections.

Most authors have performed intraorbital injections via the inferotemporal area of the lower lid.^[13,14,15,16,19,20]^ Similar to our study, Bordaberry et al^[17]^ gave intraorbital injections via both lids (superoanasal and inferotemporal) and reported the greatest decrease in CAS scores. It seems that injections at two sites through both eyelids allow better distribution of the medications in the orbit and provide a better therapeutic response. Injections from two different sites within the orbit may increase the risk of intraorbital pressure rise and may also increase the risk of retroseptal hemorrhage. Because of these considerations, we did not include patients with compressive optic neuropathy in our study, but it is interesting that some studies have demonstrated benefits from such therapy even in cases of compressive optic neuropathy.^[17,33]^

The most important complication reported after intraorbital steroid injections is intraocular pressure elevation. Even though certain studies have not reported this complication,^[14,18]^ in others, the pressure elevation was controlled with topical medications,^[16]^ but in one study this complication was seen in 30% of the cases and they had to control it in some cases by glaucoma surgery.^[13]^ In the present study, IOP rise occurred after 8.8% of the injections and all instances were readily controlled with topical medications. Another complication in our cases was subconjunctival or eyelid hematoma in 7.1% of treated eyes which improved spontaneously. A number of other complications have been reported with this modality of treatment, such as subcutaneous fat atrophy, globe perforation, central retinal artery occlusion, and toxic optic neuropathy.^[14]^ Fortunately, such catastrophic complications are rare and were not observed in our study.

At the end of the study and after resolution of disease activity, some patients required surgical intervention, which included cosmetic orbital decompression in 22.6% of eyes, repair of lid retraction in 9.7% of eyes, and strabismus surgery in 23.5% of cases. In similar studies, a need for orbital decompression was reported in 31.8–55% of eyes, eyelid repair in 64% of eyes, and strabismus surgery in 10.5–14% of cases,^[13,18]^ which was roughly similar to our series.

The major drawback to our study is the lack of a control group and the limited number of cases because of the strict inclusion criteria. In the current study, intraorbital injections were performed in the operating room; if larger studies also demonstrate the relative safety of the procedure, these injections may be used more liberally and with less expense on an outpatient basis.

In summary, intraorbital steroid injections in Graves' ophthalmopathy can help decrease orbital inflammation and are a good substitute to systemic steroids when patients become steroid resistant or dependent, or develop complications related to them.

##  Financial Support and Sponsorship

Nil.

##  Conflicts of Interest

There are no conflicts of interest.
